# Radiomic study on preoperative multi‐modal magnetic resonance images identifies IDH‐mutant TERT promoter‐mutant gliomas

**DOI:** 10.1002/cam4.5097

**Published:** 2022-09-29

**Authors:** Haoyu Wang, Shuxin Zhang, Xiang Xing, Qiang Yue, Wentao Feng, Siliang Chen, Jun Zhang, Dan Xie, Ni Chen, Yanhui Liu

**Affiliations:** ^1^ Department of Neurosurgery West China Hospital of Sichuan University Chengdu China; ^2^ Department of Neurosurgery Xinhua Hospital, Affiliated to Shanghai Jiao Tong University School of Medicine Shanghai China; ^3^ Department of Head and Neck Surgery, Sichuan Cancer Hospital and Institute, Sichuan Cancer Center, School of Medicine University of Electronic Science and Technology of China Chengdu China; ^4^ Department of Radiology West China Hospital of Sichuan University Chengdu China; ^5^ Frontier Science Center for Disease Molecular Network, State Key Laboratory of Biotherapy West China Hospital of Sichuan University Chengdu China; ^6^ Department of Pathology, West China Hospital Sichuan University Chengdu Sichuan China

**Keywords:** glioma, isocitrate dehydrogenase, magnetic resonance imaging, radiomics, telomerase reverse transcriptase

## Abstract

**Objectives:**

Gliomas with comutations of isocitrate dehydrogenase (IDH) genes and telomerase reverse transcriptase (TERT) gene promoter (IDHmut pTERTmut) show distinct biological features and respond to first‐line treatment differently in comparison with other gliomas. This study aimed to characterize the IDHmut pTERTmut gliomas in multimodal MRI using the radiomic method and establish a precise diagnostic model identifying this group of gliomas.

**Methods:**

A total of 140 patients with untreated primary gliomas were admitted between 2016 and 2020 to West China Hospital as a discovery cohort, including 22 IDHmut pTERTmut patients. Thirty‐four additional cases from a different hospital were included in the study as an independent validation cohort. A total of 3654 radiomic features were extracted from the preoperative multimodal MRI images (T1c, FLAIR, and ADC maps) and filtered in a data‐driven approach. The discovery cohort was split into training and test sets by a 4:1 ratio. A diagnostic model (multilayer perceptron classifier) for detecting the IDHmut pTERTmut gliomas was trained using an automatic machine‐learning algorithm named tree‐based pipeline optimization tool (TPOT). The most critical radiomic features in the model were identified and visualized.

**Results:**

The model achieved an area under the receiver‐operating curve (AUROC) of 0.971 (95% CI, 0.902–1.000), the sensitivity of 0.833 (95% CI, 0.333–1.000), and the specificity of 0.966 (95% CI, 0.931–1.000) in the test set. The area under the precision‐recall curve (AUCPR) was 0.754 (95% CI, 0.572–0.833) and the F1 score was 0.833 (95% CI, 0.500–1.000). In the independent validation set, the model reached 0.952 AUROC, 0.714 sensitivity, 0.963 specificity, 0.841 AUCPR, and 0.769 F1 score. MR radiomic features of the IDHmut pTERTmut gliomas represented homogenous low‐complexity texture in three modalities.

**Conclusions:**

An accurate diagnostic model was constructed for detecting IDHmut pTERTmut gliomas using multimodal radiomic features. The most important features were associated with the homogenous simple texture of IDHmut pTERTmut gliomas in MRI images transformed using Laplacian of Gaussian and wavelet filters.

## Graphical abstract

Preoperative multi‐modal magnetic resonance imaging radiomic study could distinguish IDH‐mutant TERT promoter‐mutant gliomas from other gliomas. Contrast‐enhanced T1, FLAIR, and ADC maps played an important role in detecting IDH‐mutant TERT promoter‐mutant gliomas. MR imaging of IDHmut pTERTmut gliomas showed homogenous low‐complexity texture in three modalities.

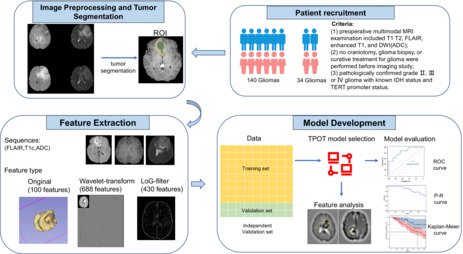

## 1 INTRODUCTION

Glioma is the most common primary malignant central nervous system tumor in adults.^1^ It can be histologically categorized into four grades (WHO I–IV). The median overall survival (OS) time of patients with glioblastoma multiforme (WHO IV) is 14–16 months. In contrast, grades II and III of glioma are relatively less aggressive, with a median survival of more than 7 years.^2^

Studies in recent decades have identified subtypes of gliomas of the same grade with different biological manifestations.^3^ Somatic missense mutations in IDH1/2 (Isocitrate dehydrogenase 1/2) genes result in the production of the mutant enzyme that converts αKG to D‐2‐hydroxyglutarate (D2HG). IDH wild‐type tumors show a higher proliferative and invasive behavior,^4^ and patients with IDH wild‐type glioma generally have a worse prognosis than those with IDH‐mutant glioma. Telomerase reverse transcriptase (TERT) keeps telomeres intact by activating telomerase, allowing cells to gain unlimited proliferation. Forty‐two percent of IDH‐mutant gliomas carry TERT promoter mutation.^5^ Two SNVs were commonly found in the TERT promoter region: C228T and C250T. They resulted in enhanced transcriptional activity of the TERT.^6^ Gliomas with both IDH and TERT promoter mutations have been shown to derive a better prognosis. Over 80% of these gliomas are oligodendrogliomas. These gliomas are amenable to nongross total resection (GTR) and are sensitive to PCV chemotherapy.^5^, ^7^ Therefore, identifying IDH‐mutant TERT promoter‐mutant (IDH‐mut TERTp‐mut) gliomas may help prognosis prediction and treatment selection.

Radiomics is a set of methodology that uses quantitative summary statistics to represent macroscopic image phenotype in a region of interest. Significant associations have been shown between radiomic features and the molecular status of tumors.^8^, ^9^ The quantitative nature of radiomics facilitates integrating information from multiple MRI sequences to produce tumor classifiers with machine‐learning techniques. Radiomic features from conventional T1 with contrast (T1c) and FLAIR MRI sequences have been exploited to predict the mutation status of IDH or TERT promoter in gliomas.^10^, ^11^ However, their performance in identifying IDH‐mut TERTp‐mut gliomas was moderate.^12^ Multimodal MRI incorporates diffuse‐weighted images (DWI), perfusion‐weighted imaging (PWI), magnetic resonance spectroscopy (MRS), and other dedicated sequences on top of conventional sequences to reveal histology, metabolism, and functional status of tumor and brain parenchyma.^13^, ^14^ DWI characterizes the Brownian motion of water molecules, reflecting the tumor cell density.^15^ Apparent diffusion coefficient (ADC) images, or ADC maps, show diffusion more specifically than conventional DWI by eliminating the T2 weighting in conventional DWI.

In contrast to DWI, the standard grayscale of ADC images represents larger diffuse amplitudes as brighter.^16^ High‐grade gliomas show hypointense signals on ADC maps.^17^ The mean ADC values of IDH‐wt gliomas were significantly lower compared with IDH‐mut gliomas.^18^ However, accurate preoperative diagnosis of TERTp status using mean ADC values of the tumor region remains a challenge.^19^ We hypothesize that the ADC map can be combined with conventional T1 and FLAIR to capture a more comprehensive profile of IDH‐mut TERTp‐mut gliomas and build an accurate classifier.

In the present study, we extracted radiomic features from multimodal imaging sequences (T1c, FLAIR, and ADC maps) and identified diagnostic features that specifically detect IDHmut pTERTmut comutant gliomas in a data‐driven approach. We also attempted to characterize the comutants by interpolating the important radiomic features and predicting patient outcomes using the output of the radiomic model.

## 2 MATERIALS AND METHODS

### 2.1 Patients

A case–control study was designed to evaluate the application of preoperative multimodal radiomic in identifying IDH‐mutant TERT promoter‐mutant gliomas. All included patients met the following criteria: (1) preoperative multimodal MRI examination included T1, T2, FLAIR, enhanced T1, and DWI; (2) no craniotomy, glioma biopsy, or curative treatment for glioma was performed before the imaging study; (3) pathologically confirmed grade II, III, or IV glioma with known IDH status and TERT promoter status (Supplementary Methods). The collection of MRI data was compliant with the principles of the Declaration of Helsinki. Of all the patients, 22 in the discovery cohort and 7 in the validation cohort had IDH‐mut TERTp‐mut gliomas.

### 2.2 Image preprocessing and tumor segmentation

The T1, T2, FLAIR, and enhanced T1 image was performed with a German‐made Siemens 3.0 T MR scanner (Supplementary Methods). The ADC maps were obtained by automatic processing of DWI images by the syngo MR image workstation. T1, T2, FLAIR, enhanced T1, and ADC images were first had the cranium removed using the SwissSkullStripper module in 3DSlicer software.^20^ Next, we registered T1, T2, FLAIR, and ADC sequences with the enhanced T1 sequence by the General Registration (BRAINS) module, respectively.

The tumor segmentation was performed with a process combining automatic segmentation and manual correction. The automatic segmentation protocol is based on a set of niftynet and TensorFlow applications, laid out in python 3.6.^21^ It can automatically segment the tumor region based on four sequence images: T1, T2, FLAIR, and T1c. After coregistration, T1, T2, FLAIR, and enhanced T1 images were input into the automatic segmentation model to obtain the ROI mask containing three parts: tumor necrosis, solid, and edema.^22^ The original automatic segmentation mask was then input into ITK‐snap^23^ software and manually corrected based on multimodal images by a radiologist (with 20 years of experience in neuro‐oncology) to remove the mis‐segmentations (Supplementary Methods).

### 2.3 Radiomic features extraction

For further radiomic analysis, radiomic features were extracted using a python package named pyradiomics^24^ (Pyradiomics Library, version 2.2.0). In the pyradiomics pipeline, the grayscale values of the images were first normalized by centering at the image mean and scaling with the standard deviation. Next, the images and segment masks were resampled to a specified pixel spacing ([1,1,1]), using a Bspline interpolator. Pyradiomics can extract 1218 features per sequence including 2D and 3D intensity, shape, and texture features from raw voxels, LoG filter (Laplacian of Gaussian), and wavelet transformed voxels (Table S2). A total of 3654 (1218 × 3) features were extracted from the T1c, FLAIR, and ADC map sequences (1218 features include as follows: (1) 18 first‐order features, (2) 14 shape features, (3) 68 textural features (4), 430 LoG filter features, and (5) 688 wavelet features).

In LoG images, regions of rapid intensity change were highlighted. Details of edge texture were better seen.^25^ In wavelet transformed images, the wavelet filter decomposed the signal of each dimension into high‐frequency components (H) and low‐frequency components (L). 3D images were decomposed at each dimension to produce a total of eight subimages (2*2*2), one of which corresponded to the smooth version (LLL), and the remaining seven correspond to the detailed version (LLH, LHL, LHH, HLL, HLH, HHL, HHL, and HHH).^26^ Utilizing pyradiomics, cv2, and pywt package on python 3.6, we performed LoG filter and wavelet transform processing on screenshots of the maximum cross sections of different sequences of tumors to compare the extracted relevant features in different groups.

### 2.4 Model development

To evaluate the performance of multimodal features on the model predictive capacity, we organized all features into one multimodal feature set, which contained features extracted from T1 enhanced, FLAIR, and ADC sequences. The discovery data set was divided into the training set and test set in the ratio of 4:1. The training set features were subjected to feature selection by least absolute shrinkage and selection operator (LASSO) feature dimensionality reduction. Before feature reduction by LASSO, all extracted features were normalized using Z‐score normalization. The minimum value of λ and the minimum value plus one MSE were determined after 10‐fold validation, so the features with non‐zero coefficients were obtained from the selected λ screening (the minimum λ). Next, each training data set after feature dimensionality reduction was input to a tree‐based pipeline optimization tool (TPOT) for model development (http://epistasislab.github.io/tpot/). TPOT is an automated machine‐learning (AutoML) algorithm designed to search for the optimized machine‐learning pipeline and minimize the need for human interference in this process. TPOT uses a genetic algorithm for feature and model selection to optimize models automatically, features and hyperparameters, and generate the final optimal model.

**FIGURE 1:**
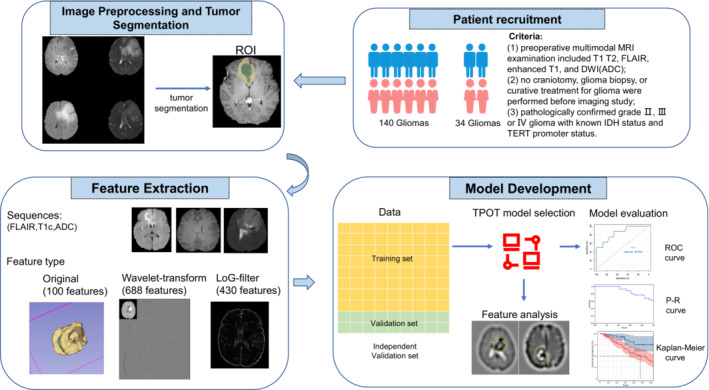
An overview of the current study. The workflow of the study included patient recruitment, image preprocessing and tumor segmentation, feature extraction, and model development. The region of interest (ROI) of the glioma was automatically segmented from T1Gd (T1C), FLAIR, T2, and T1 using the open‐source NiftyNet platform. The radiomic features of three sequences were extracted from the segmented ROI in original images, Laplacian‐of‐Gaussian (LoG) filter images, and wavelet filter images. After the LASSO feature dimensionality reduction, the entire data set was split into a training set (yellow color), a test set (green color), and an independent validation set (orange color). The training set was used to search and train an optimal model using TPOT. The optimal model was evaluated on the test set and independent validation set. Then, we attempted to interpret key diagnostic features for IDHmut pTERTmut glioma. Finally, we assessed the survival curves in different subgroups.

TPOT parameters were selected: generation number, 50; population size, 100; and 10‐fold internal cross‐validation. Generation refers to the number of iterations, and population refers to the number of primaries (i.e., the number of pipelines generated by the primaries). One iteration round will cross‐mutate the previous round's individuals to generate an equal number of new individuals. Then, TPOT scores the old and new individuals simultaneously to select the best pipeline with the same number of primaries into the next round of iteration. After a specified number of iterations, the best pipeline was selected. In this pipeline, ANOVA (analysis of variance) was used for further feature selection. The classification model is a multilayer perceptron (MLP) classifier that was a fully connected class of feedforward artificial neural network (ANN). Two hundred random training/test set splits were used to validate the reproducibility of the model performance. Subsequently, the predictive performance of our model was examined in the independent validation set. The workflow is shown in Figure 1.

After developing the optimal model, with the aid of coefficients of lasso dimensionality reduction, we measured the weights of the features via the ELI5 package (ELI5 Library, version 0.11.0) in python 3.6 to score the importance of features and selected key features from multimodal sequences.

### 2.5 Statistical analysis

Except for age, the baseline characteristics of the two groups (IDHmut/pTERTmut and non‐IDHmut/pTERTmut) in each data set (discovery set and independent validation set) were compared using the chi‐square test. Mann–Whitney test was used for baseline difference in age and to assess the differences in feature value between the double mutations group and the nondual mutation group. Kruskal–Wallis test was used for accessing statistical differences of features across subgroups of patients (IDHmut pTERTmut, IDHmut pTERTwt, IDHwt pTERTmut, and IDHwt pTERTwt). Receiver‐operating characteristic (ROC) and precision–recall (P‐R) analyses were conducted to evaluate the performance of models in the prediction of comutation status. The predictive ability of the prediction model was assessed by specificity, sensitivity, accuracy, precision, recall, F1 score, PRAUC, and AUC value. The 95% CIs of these performance metrics using 200 random training/test set splits. The log‐rank test was used to test for differences in the overall survival of patients. A 95% CI was set, and *p* < 0.05 was considered statistically significant. The raw data of radiomic features are attached to Table S5.

## 3 RESULTS

### 3.1 Clinical characteristics

According to the inclusion criteria (Figure 1), a total of 140 patients with untreated primary glioma admitted between 2016 and 2020 in the Department of Neurosurgery, West China Hospital were retrospectively included as the discovery cohort. An additional cohort consisting of 34 primary glioma patients from West China Shangjin Hospital was collected as the independent validation data set (Table 1). All patients received preoperative multimodal MRI examination (T1c + FLAIR+ADC). The number of patients in two data sets with IDHmut pTERTmut glioma was 22/140 (15.7%) and 7/34(20.6%). There was no significant difference in gender between the two groups of IDH/pTERT glioma. The IDHmut pTERTmut glioma patients were significantly younger than the other patients in both the discovery set and independent validation set (*p* = 0.02 and *p* = 0.02). Most IDHmut pTERTmut gliomas (28/29) were lower‐grade gliomas (WHO II/III). The clinical and statistical results of the study are summarized in Table 1.

**TABLE 1:** Patient demographics and tumor genotypes

	Training and test set		*p* value	Independent validation set		*p* value	*p* value
	IDHmut/pTERTmut (*N* = 22)	Non‐IDHmut/pTERTmut (*N* = 118)		IDHmut/pTERTmut (*N* = 7)	Non‐IDHmut/pTERTmut (*N* = 27)		
Age, median (IQR), years	46 (14.8)	51 (21.5)	0.02^a^	40 (8.2)	53 (12.2)	0.02^a^	0.22^a^
Sex			0.48^b^			0.25^b^	0.91^b^
Male	9 (41%)	79 (67%)		3 (43%)	18 (67%)		
Female	13 (59%)	39 (33%)		4 (57%)	9 (33%)		
Grade			<0.001^b^			<0.001^b^	0.25^b^
Lower‐grade (WHO II/III)	21 (95.5%)	48 (40.7%)		7 (100%)	6 (22.2%)		
WHO II	16	32		5	5		
WHO III	5	16		2	1		
High grade (WHO IV)	1 (4.5%)	70 (59.3%)		0 (0%)	21 (77.8%)		
IDH							
Mutant	22	26		7	5		
Wild‐type	0	92		0	22		
TERT promoter							
Mutant	22	53		7	15		
Wild‐type	0	65		0	12		
1p19q							
Noncodeletion	1	23		0	2		
Codeletion	17	2		7	1		

Abbreviations: IDHmut, isocitrate dehydrogenase mutation; pTERTmut, Telomerase reverse transcriptase promoter‐mutant.

^a^ Mann–Whitney test.

^b^ Chi‐square test.

### 3.2 Identification of potential IDHmut pTERTmut Glioma‐Specific radiomic features

**FIGURE 2:**
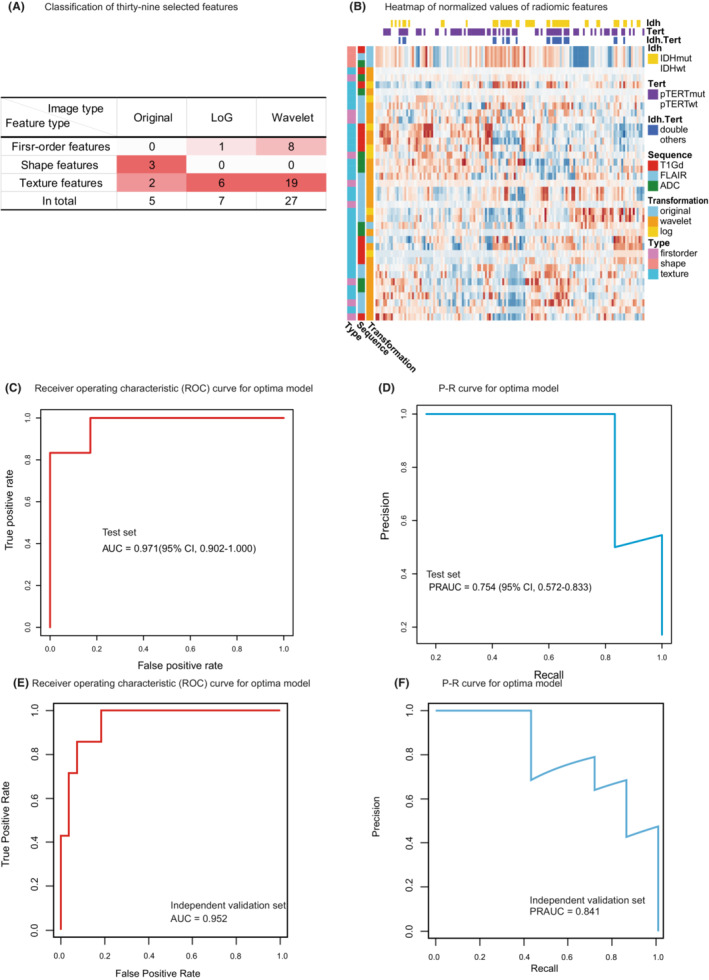
Selected Features and Performance of The Prediction Model for Identifying IDH‐mutant TERT promoter‐mutant Gliomas. (A) The least absolute shrinkage and selection operator (Lasso) binary logistic regression model identified 39 radiomic features specific to IDHmut pTERTmut gliomas from a total of 3654 features. (B) Samples and features were clustered using unsupervised hierarchical clustering. (C, D), Performance of the prediction model (receiver operating characteristic [ROC] curve and P–R curve analysis). (E, F), Performance of the prediction model in the independent validation set (ROC curve and P–R curve analysis). Abbreviations: LoG, Laplacian of Gaussian.

We identified the radiomic features specific to IDHmut pTERTmut gliomas in a data‐driven approach. After extracting 3654 features from the automatically segmented tumor, we selected potential predictive features for IDHmut pTERTmut gliomas from the multimodal feature set, by training lasso regression models with a series of λ. Nonzero coefficient features in the model with minimum mean square cross‐validation error were defined as specific to IDHmut pTERTmut gliomas (Figure S1A,B). Thirty‐nine IDHmut pTERTmut glioma‐specific radiomic features were selected (Figure 2A). Unsupervised clustering of samples using these specific features revealed heterogeneity among IDHmut pTERTmut gliomas. Clustering features found a higher correlation between the extracted features from original images than those extracted from LoG and wavelet transformed images (Figure 2B, Figure S2).

### 3.3 Detection of IDHmut pTERTmut gliomas using multimodal radiomic features

To understand if the multimodal radiomic features could be used to distinguish IDHmut pTERTmut gliomas from other gliomas, we built a diagnostic model using an autoML algorithm with 80% of the patients' data (112) as the training set. We evaluated the performance of the model with the rest 20% of the cohort (28). The autoML algorithm searched through a series of modeling pipelines combining different data feature reduction methods and models to find the best pipeline for detecting IDH‐mut TERT promoter‐mut gliomas. The best pipeline was found to be a pipeline that consisted of selecting the top 4% ANOVA F value between label/feature, followed by appending the count of zero‐value features for each sample as an additional feature, and finally fitting a multilayer perceptron classifier (MLP classifier). The pipeline reached a sensitivity of 0.833 (95% CI, 0.333–1.000) at a specificity of 0.966 (95% CI, 0.931–1.000) in the test set. The overall accuracy was 0.943 (95% CI, 0.886–1.000). The area under the receiver‐operatoring curve (AUROC) was 0.971 (95% CI, 0.902–1.000, Figure 2C). Given the relatively small proportion of IDHmut pTERTmut gliomas, we also conducted a precision–recall analysis. The precision of this pipeline was 0.833 (95% CI, 0.667–1.000). The area under the precision–recall curve (AUCPR) was 0.754 (95% CI, 0.572–0.833, Figure 2D), and the F1‐score was 0.833 (95% CI, 0.500–1.000). Here, we reported the 95% CIs of these performance metrics using 200 random training/test set splits to show the reproducibility of the results. Furthermore, the predictive performance of the model was evaluated in the independent validation set. The overall accuracy, sensitivity, and specificity were 0.912, 0.714, and 0.963 The AUROC was 0.952 (Figure 2E). The precision of this pipeline was 0.833. The AUCPR was 0.841 (Figure 2F) and the F1 score was 0.769. These results suggested that the specific multimodal radiomic features could build robust diagnostic tools for detecting IDHmut pTERTmut gliomas.

### 3.4 Prognostic value of the radiomic model output

In current practice, the aggressiveness of suspected glioma is usually assessed using enhancement or nonenhancement.^27^ Most malignant GBMs present in T1c images as an enhancing tumor, while less‐aggressive LGGs usually present as a nonenhancing tumor, but the exceptions are common.^28^ Interestingly, in the test set, the OS for patients with contrast‐enhanced (CE) tumors appeared to be better than those with non‐CE tumors, although the difference was nonsignificant (*p* = 0.091, Figure 5A). To understand if the radiomic model could provide more accurate prognostic information, we analyzed the OS of patients with different predicted comutation statuses. In patients with predicted non‐comutation gliomas, six patients died, while none of the patients with comutation died during our follow‐up (*p* = 0.29, Figure 5B).

### 3.5 Interpreting diagnostic radiomic features of IDHmut pTERTmut Glioma

**FIGURE 3:**
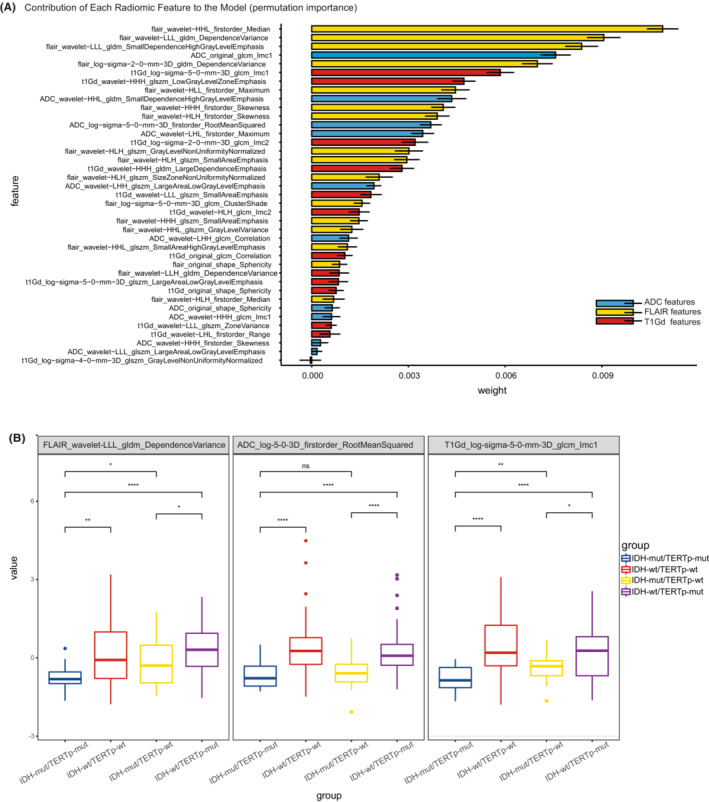
Feature importance and comparison of key feature values. (A) The contribution of IDHmut pTERTmut glioma‐specific radiomic features to the diagnostic model was estimated using the ELI5 package. The error bars in the figures were shown as standard deviations of weights in 200 random training/test set splits. (B) Boxplot of feature values. The value of key radiomic features in each sequence among IDHmut/pTERTmut and other groups were compared using the Mann–Whitney test (*p value <0.05, **p value <0.01, ***p value <0.001, ****p value <0.0001). Abbreviations: GLCM, gray‐level co‐occurrence matrix; GLDM, gray level dependence matrix; GLSZM, gray‐level size zone matrix; LoG, Laplacian of Gaussian.

**FIGURE 4:**
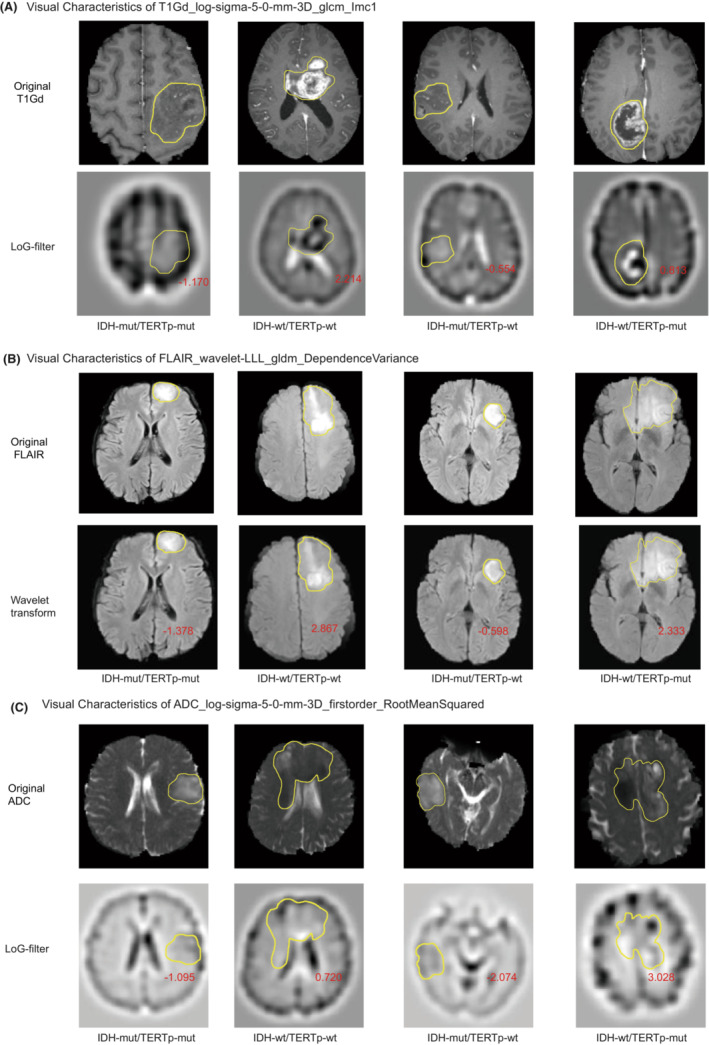
The Appearance of The MR Image Corresponds to Three Key Radiomic Features in IDH‐mutant TERT promoter‐mutant and Other Gliomas. (A–C) suggested that the IDHmut pTERTmut gliomas showed homogenous low‐complexity texture in T1Gd (T1C), FLAIR, and ADC sequences. Yellow lines indicated the region of gliomas. Red numbers denoted the feature values of the region of interest (ROI). Abbreviations: Imc1: Informational Measure of Correlation.

Despite the high performances of multivariate radiomic models in solving controlled classification tasks, the interpretability and transparency of the diagnostic results are still desirable for both clinicians and patients. In terms of radiological characteristics, there was a higher percentage of cases were marked/avid or minimal/mild enhancing in non‐IDHmut/pTERTmut group (*p* < 0.001). Although there was no significant difference in T2/FLAIR mismatch sign between the two groups (Table S3). We attempted to interpret the result of the IDHmut pTERTmut glioma detection model by evaluating the most important diagnostic radiomic features. We estimated the contribution of each radiomic feature to the model by calculating its permutation importance which was represented by the mean decrease in model accuracy when values of the feature were permuted among samples. We found that IDHmut pTERTmut glioma‐specific radiomic features from all three modalities contributed to the diagnostic model (Figure 3A). Among the top 10 most important features, six were extracted from FLAIR images, two from ADC maps, and two from T1c images. The most important features were FLAIR_wavelet‐HHL_firstorder_Median, T1Gd_log‐sigma‐5‐0‐mm‐3D_glcm_Imc1, and ADC_original_glcm_Imc1 in each modality. Though FLAIR_wavelet‐HHL_firstorder_Median and ADC_original_glcm_Imc1 ranked the most important feature in their respective modality, these features' difference between molecular subgroups was non‐significant (adjusted *p* = 0.14 and *p* = 0.34, Table S4). Nonetheless, among the top features, FLAIR_wavelet‐LLL_gldm_DependenceVariance and ADC_log‐sigma‐5‐0‐mm‐3D_firstorder_RootMeanSquared were significantly different between IDHmut pTERTmut gliomas and other gliomas (adjusted *p* < 0.001, Figure 3B, Table.S4). To visualize these diagnostic radiomic features, we transformed the original images with the corresponding filters and compared the characteristics of different glioma groups. Intuitively, the IDHmut pTERTmut gliomas showed homogenous low‐complexity texture in three modalities (Figure 4).

**FIGURE 5:**
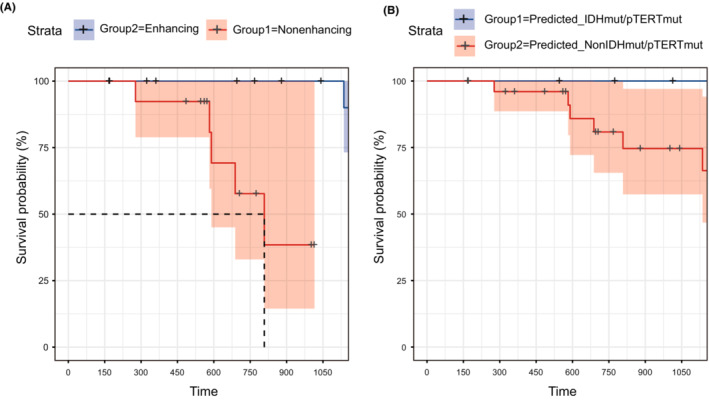
Kaplan–Meier analysis of test set patients while substratifying by predicted‐IDHmut/pTERTmut or enhancing quality. (A) OS for patients with enhancement and patients without enhancement in T1C image. (B) OS for patients with predicted‐IDHmut/pTERTmut and patients with predicted‐NonIDHmut/pTERTmut.

The diagnostic radiomic features in ADC and T1c images were extracted from LoG‐transformed images with a sigma of 5.0 mm. The LoG filter enhances areas with rapidly changing intensities.^25^ Intuitively, the root mean squared of LoG ADC maps in the tumor region quantifies the average abruptness of diffusion changes. Therefore, lower ADC_log‐sigma‐5‐0‐mm‐3D_firstorder_RootMeanSquared values in the IDHmut pTERTmut gliomas may represent a more homogenous diffusion in the tumor, while abrupt changes in ADC intensity could reflect the presence of non‐liquefied necrotic tissue found in more aggressive glioma groups (Figures 3B, 4C).

Gray Level Cooccurrence Matrix (GLCM) counts the number of gray level intensities combinations in connected voxels.^29^ The Informational Measure of Correlation 1 (IMC1) of the GLCM measures enrichment of any particular intensity combinations and correlated with the complexity of image texture (images with repeating predictable texture contain fewer intensity combinations, thus yielding an IMC1 close to −1, while those with complex unpredictable texture contain widely dispersed intensity combinations and produces an IMC close to 0). For the LoG T1c image, the low GLCM IMC1 in IDHmut pTERTmut gliomas indicated low complexity texture in the tumor region (Figures 3B, 4A).

Wavelet decomposition separates image information using a sequence of wavelet functions from higher frequency wavelets to lower frequency ones.^26^ The relatively subtle information approximated by the higher frequency wavelet function is captured by the high‐pass filter, while the remaining information is captured by the low‐pass filter and can be further decomposed using wavelet functions with lower frequencies. The LLL wavelet transformed FLAIR images had high‐frequency signals removed and could therefore be considered a smoothed FLAIR image. The Gray Level Dependence Matrix (GLDM) counts the number of patterns formed by connected voxels with similar intensity.^30^ The dependence variance of GLDM describes the diversity of patterns with higher values representing more diverse patterns in an image. IDHmut pTERTmut gliomas showed low GLDM dependence variance in LLL wavelet transformed FLAIR. This could also be considered a sign of its homogenous texture (Figures 3B and 4B).

Upon inspection, the differences between IDHmut pTERTmut and IDHmut pTERTwt gliomas were not as obvious as those between IDHmut pTERTmut and IDH wild‐types (Figure 3B). Consequently, we further analyzed significantly differential radiomic features between the two IDH‐mutant groups. We found FLAIR_wavelet‐HLH_glszm_GrayLevelNonUniformityNormalized, t1Gd_wavelet‐LLL_glszm_SmallAreaEmphasis, and ADC_wavelet‐HHH_glcm_Imc1 had the smallest adjusted *p* values in their respective modality (Figure S4, Table S4). The FLAIR and ADC differential features could be interpreted into simple or uniform intensity signals in the high‐pass wavelet filter transformed images, while the t1Gd_wavelet‐LLL_glszm_SmallAreaEmphasis indicated fewer fine patterns (small‐size connected voxel zones) in the smoothed T1c image (Figure S5A).

## 4 DISCUSSION

The outcomes of adult gliomas are diverse. While patients with highly aggressive IDH wild‐type gliomas have an abysmal prognosis, those with IDH gene and TERT gene promoter comutant tumors receive much greater benefit from current treatments.^31^, ^32^ Over 80% IDHmut pTERTmut gliomas are oligodendrogliomas characterized by codeletion of chromosome arms 1p and 19q.^5^ This group of gliomas has been shown to derive similar benefits from non‐GTR compared with GTR and respond to PCV chemotherapy.^33^ This study attempted to develop a noninvasive method to detect IDH‐mutant TERT promoter‐mutant gliomas using preoperative multimodal MRI. We first extracted quantitative features using radiomic analysis and selected those most relevant to IDH‐mutant TERT promoter‐mutant gliomas in a data‐driven approach. Consequently, we identified a set of diagnostic radiomic features that depicted the homogenous simple texture of these gliomas. Since a higher proportion of strongly enhancing cases was found in the non‐IDHmut/pTERTmut group than IDHmut/pTERTmut group in the discovery set (Table S3), we attempted to use enhancement quality as a factor for prognostic stratification in the test set. The K‐M curves showed that the enhancement quality was not a prognostic stratification indicator for the glioma patients in the test set. With the model, we attempted to verify the significance of molecular subgroups, predicted by the model, in the prognostic stratification of glioma patients. Although not statistically significant, there was a worsening trend in overall survival in the predicted_NonIDHmut/pTERTmut group.

Time savings was an important benefit of using TPOT compared with manual annotation and model parameters searching. With the development of deep‐learning technology and the construction of related glioma MRI data sets (e.g., Brats competition), glioma's automatic tumor segmentation model has become more and more mature.^34^ A recent study reported a deep‐learning approach for tumor segmentation and grading, combining CNNs, transfer learning based on a pretrained model, a fully connected classifier, and the dice similarity coefficient (DSC) score reach 0.84.^35^ However, the drawback was that for diffuse or multiple lesions, automated segmentation results were not entirely satisfactory. Experienced radiologists were needed to correct and proofread the segmented tumor images. TPOT, the result of the autoML research, integrates multiple models (random forest, XGBOOST, etc.) developing pipelines, which can most effectively perform feature engineering and model building. Prior studies have confirmed that the model from TPOT has better performance than from AdaBoost or random forest.^36^, ^37^ However, the disadvantage of TPOT is that a large number of feature inputs can reduce the program's speed. Therefore, advanced feature selection such as LASSO feature dimensionality reduction was used in our study, which can significantly accelerate the running speed of TPOT.

Previous radiomic studies focused on the diagnosis and prognosis of glioma for prediction.^38^ On the one hand, progress has been made in the prediction of a single genotype. Based on the radiomic features extracted from DWI, PWI, and conventional MRI, a logistic regression classifier was used for predicting the IDH status of LGGs. The AUC of the model reached 0.795 in the validation set.^39^ Another study showed similar results, using conventional MRI features and arterial spin labeling (ASL) image features to develop a linear SVM model to predict IDH mutations in gliomas, and the AUC reached 0.823.^11^ As for some radiomic studies aimed at pTERT mutations, two prior studies evaluate the performance of random forest and linear SVM models in predicting pTERT mutations based on conventional MRI features. The results of the two studies showed AUC reached 0.827 and 0.845, respectively.^10^, ^40^

On the other hand, some studies addressed combining subtypes of gliomas for predictions. A lasso regression model was developed for predicting IDH and pTERT mutations based on the conventional MRI radiomics analysis. The overall diagnostic accuracy in the validation set reached 0.56.^12^ Logistic regression models were built to predict IDH‐wild‐type TERTp‐mutation high‐grade gliomas using pretreatment dynamic [^18^ F]FET PET radiomics methods. The AUC of the best model reached 0.82 with nine selected features.^41^ The application of CNNs in medical image analysis has been growing rapidly in recent years. A TERT promoter mutation classifier's accuracy reached 84.0 ± 9.3% using CNN base texture features.^42^ A gradient boosting model was trained using the textual and shape features from the LoG‐filtered T1c image for predicting the co‐occurrence of IDHmut and MGMTmet in gliomas, and the AUC reached 0.951.^43^ It was noteworthy that the texture feature set had a large weighting in many prediction models, which corroborated our study. Texture features accounted for above 80% of the model‐selected features in a radiomic study for IDH prediction.^11^ Twelve features incorporated into the pTERT prediction model by Fang et al. were texture features and wavelet transform features.^10^ Nine features finally incorporated into the model of pTERT prediction study contained eight texture features, and six texture features were the same or similar to ours.^40^ Among them, three T1c features (correlation, gray‐level nonuniformity normalized, and gray‐level variance) were the same as our selected T1c features, and three T2 features (gray‐level variance, large dependence on high gray‐level emphasis, and small dependence on high gray‐level emphasis) were similar to our selected FLAIR features. Textural features of conventional MRI also showed nice prognostic performance (C‐index, 0.798) in LGG patients,^44^ most of them were wavelet‐transformed textural features (27/29), and six wavelet features were identical to our study, including our key feature (FLAIR_wavelet_LLL_gldm_DependenceVariance). In our study, the texture features contributed the most to the model. There are two possible reasons for this result. First, texture features took the largest proportion in the radiomics feature set. Second, texture features convoluted the numerous internal details of ROI and contains more “microscopic” information which is more resilient to the heterogenous growth and invasion patterns of gliomas.^45^, ^46^

In addition to the key features between IDHmut pTERTmut and IDHmut pTERTwt gliomas, the interpretation of significantly differential radiomic feature between the two IDH‐mutant groups is also of interest. The information captured in high‐pass wavelet filters has often been regarded as a noise component of the image. However, the high‐pass wavelet filter transformed ADC maps showed a significant difference in GLCM IMC1 between IDHmut pTERTmut and IDHmut pTERTwt gliomas. Lower ADC_wavelet‐HHH_glcm_Imc1 of IDHmut pTERTmut gliomas turned out to represent a lack of high‐frequency component (Figure S5C).

Gray‐Level Size Zone Matrix (GLSZM) counts connected zones (patterns) of various sizes (number of voxels).^47^ The normalized gray‐level nonuniformity of GLSZM is positively correlated with the variation in the sizes of zones. Lower nonuniformity (higher uniformity) in sizes of high‐frequency intensity zones in FLAIR images of IDHmut pTERTmut gliomas may indicate a simpler texture pattern in the high‐frequency of their FLAIR image. In smoothed wavelet‐LLL T1c images (Figure S5A), the IDHmut pTERTmut gliomas demonstrated fewer small zones than IDHmut pTERTwt gliomas (which represent finer texture patterns in an image).

A few limitations should be noticed when interpreting the results of this study. First, although we evaluated the robustness of our model using cross‐validation and independent external validation, the size of the data set needs to be expanded to obtain more powerful conclusions. Second, we omitted PWI MRS because postprocessing of these data types was not well compatible with the radiomic analysis, and we did not acquire these sequences from every included subject. Future studies may consider combining these data with extracted radiomic features to expand the MRI‐based glioma classifier. Third, because of our limited follow‐up time, none of the patients with predicted IDHmut pTERTmut did reach the survival endpoint, which impaired the significance of the statistical test comparing the two survival curves. However, the prediction made by the radiomic model still appeared to outperform conventional practice. Finally, the steep learning curve about the data‐processing techniques, the indirect manifestations of radiomic features, and the complexity of the classification model could prevent easy access to the analysis results for clinicians, especially in urgent situations. While loose associations could be established between radiomic feature values and trends in the visual characteristics of IDHmut pTERTmut gliomas, future engineering work is still necessary to better integrate the relevant information into the clinical user interface.

In conclusion, we identified specific radiomic features that detect gliomas with IDH and TERT promoter mutations from multi‐modal MRI that consisted of T1c, FLAIR, and ADC maps. We showed the performance of an automatically trained diagnostic model for this particular group of gliomas based on these features. We demonstrated the most relevant radiomic features were associated with the homogenous simple texture of IDHmut pTERTmut gliomas in MRI images transformed using Laplacian of Gaussian and wavelet filters.

## FUNDING INFORMATION

This study has received funding from the Clinical Research Innovation Project, West China Hospital, Sichuan University (19HXCX009 to Y.L.), Sichuan Provincial Science and Technology Support Plan (grant nos. 2017SZ0006 to Y.L. and 2020YFS0051 to D. Xie), and Post‐Doctor Research Project, West China Hospital, Sichuan University (20HXBH035 to S. Zhang).

## CONFLICT OF INTEREST

The authors declare that they have no potential conflict of interest.

## ETHICS STATEMENT

The collection of MRI data was compliant with the principles of the Declaration of Helsinki. The study protocols were approved by the West China Hospital Institutional Review Board. Informed consent was obtained from each patient included in the study.

## Supporting information


Table S1
Table S2
Table S3
Table S4
Figure S1
Figure S2
Figure S3
Figure S4
Figure S5

Table S5

## Data Availability

The raw data of radiomic features are attached to Table S5. The MRI images are not publicly available due to privacy or ethical restrictions. Further information is available from the corresponding author upon reasonable request.
