# Supplementary material for: Radiomic study on preoperative multi‐modal magnetic resonance images identifies IDH‐mutant TERT promoter‐mutant gliomas
Source: Cancer Med. 2022 Sep 29;12(3):2524–37. doi: 10.1002/cam4.5097 (PMC9939206; doi:10.1002/cam4.5097)
Supplement: Supplementary file 1 — Table S1 Table S2 Table S3 Table S4 Figure S1 Figure S2 Figure S3 Figure S4 Figure S5 [file CAM4-12-2524-s001.docx]

Supporting Information

Methods. Supplementary Methods

Table. S1. Parameters of Magnetic Resonance Imaging (MRI) images

Table. S2. The list of all original features

Table. S3. Radiological characteristics of the two groups of IDH/pTERT glioma

Table. S4. Thirty-nine selected features after LASSO feature dimensionality reduction

Table. S5. The raw data of radiomic features

Figure. S1. Feature selection by LASSO feature dimensionality reduction

Figure. S2. Correlation between features

Figure. S3. Pearson correlation of features before and after LASSO

Figure. S4. A boxplot comparing the difference in key radiomics features between IDHmut/pTERTmut and IDHmut/pTERTwt groups

Figure. S5. The appearance of MR image corresponds to 3 radiomics features in IDHmut/pTERTmut and IDHmut/pTERTwt gliomas

**Supplementary Methods**

**1. Acquisition of Information on IDH and TERT promoter Statuses：**

Polymerase chain reaction (PCR) followed by Sanger sequencing for genomic regions spanning codon 132 of IDH1, codon 172 of IDH2, and the TERT promoter (chr5:1295228,1295250 on hg19 genome) were performed for each glioma to determine their mutation status.

For histological assessment, if the two neuropathologists failed to agree on the initial diagnosis, a multidisciplinary board formed by senior pathologists, radiologists, oncologists, and neurosurgeons would review the case to make one consensus conclusion. With this convention, no case in the present study received discordant diagnosis.

**2. Image Preprocessing and Tumor Segmentation**

**2.1 Image Preprocessing**

T1, T2, FLAIR, enhanced T1, and ADC images were first had the cranium removed using the SwissSkullStripper module in 3DSlicer software. Next, we registered T1, T2, FLAIR, and ADC sequences with the enhanced T1 sequence by the General Registration (BRAINS) module, respectively.

**2.2 Tumor Segmentation**

A region of interest (ROI) encompassing whole tumors was segmented using the protocol based on a set of niftynet and TensorFlow applications, laid out in python 3.6. It can automatically segment the tumor region based on four sequence images: T1, T2, FLAIR, and T1c. After co-registration, T1, T2, FLAIR, and enhanced T1 images were input into the automatic segmentation model to obtain the ROI mask containing three parts: tumor necrosis, solid, and edema.

**2.3 Remove the mis-segmentations**


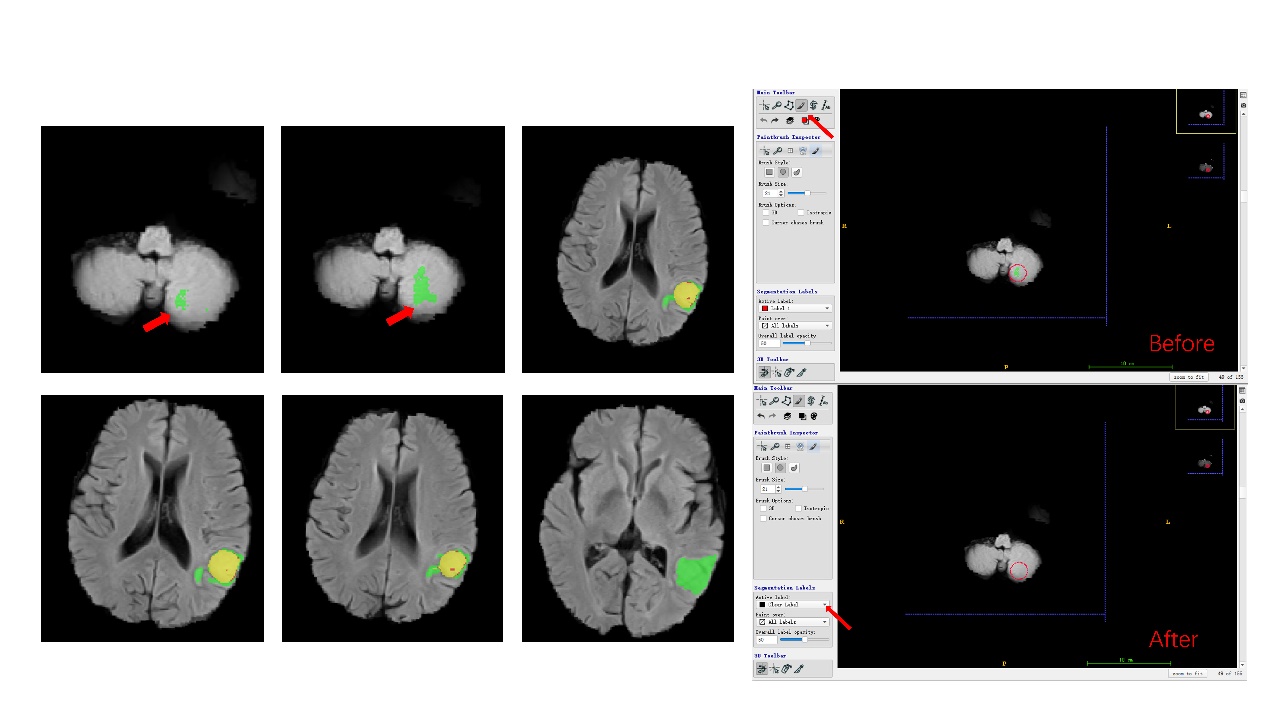


Step 1: Open “ITK-SNAP” and load the FLAIR images, then click “Segmentation” button to load automatic segmentation mask (color region). Red arrows highlight several mis-segmentations.

Step 2: Click “brush” and chose “clear label” from segmentation labels (red arrow) select the region that needs to be removed and click the right mouse button to clear the mask in the area. Finally, click “Save Segmentation image” button to save the final mask.

**3. Radiomics Feature Extraction**

Pyradiomics can extract 1218 features per sequence, including 2D and 3D intensity, shape, and texture features from raw voxels, LoG-filter (Laplacian of Gaussian), and wavelet transformed voxels (Supplementary Table S1). A total of 3654 (1218×3) features were extracted from the T1c, FLAIR, and ADC map sequences (1218 features include: 1) 18 first-order features; 2) 14 shape features; 3) 68 textural features 4); 430 LoG filter features; 5) 688 wavelet features).

In LoG images, regions of rapid intensity change were highlighted. Details of edge texture were better-seen[1]. In wavelet transformed images, the wavelet filter decomposed the signal of each dimension into high-frequency components (H) and low-frequency components (L). 3D images were decomposed at each dimension to produce a total of 8 sub-images (2*2*2), one of which corresponded to the smooth version (LLL), and the remaining seven correspond to the detailed version (LLH, LHL, LHH, HLL, HLH, HHL, HHL, HHH)[2]. Utilizing pyradiomics, cv2, and pywt package on python 3.6, we performed LoG filter, and wavelet transform processing on screenshots of the maximum cross-sections of different sequences of tumors to compare the extracted relevant features in different groups.

**4. Model Development**

**4.1 Least absolute shrinkage and selection operator (LASSO) feature dimensionality reduction**

The training set features were subjected to feature selection by least absolute shrinkage and selection operator (LASSO) feature dimensionality reduction. The minimum value of λ and the minimum value plus one MSE were determined after 10-fold validation, so the features with non-zero coefficients were obtained from the selected λ screening (The minimum λ).

**4.2 Tree-based pipeline optimization tool (TPOT)**

TPOT parameters were selected: generation number, 50; population size, 100; and 10-fold internal cross-validation. Generation refers to the number of iterations, and population refers to the number of primaries (i.e., the number of pipelines generated by the primaries). One iteration round will cross-mutate the previous round's individuals to generate an equal number of new individuals. Then, TPOT scores the old and new individuals simultaneously to select the best pipeline with the same number of primaries into the next round of iteration. After a specified number of iterations, the best pipeline was selected by TPOT.

**Table. S1. Parameters of Magnetic Resonance Imaging (MRI) images:**

|  | TR | TE | Layer thickness | Matrix | scan field FOV | Flip angel | Resolution | Others |
| --- | --- | --- | --- | --- | --- | --- | --- | --- |
| T1W1 | 1630ms | 2.3ms | 1mm | 256×256 | 230*230mm | 8° | (0.8984,0.8984,1) |  |
| Gd-T1WI^*^ | 1540 ms | 2.4ms | 1mm | 256×256 | 230*230mm | 8° | (0.8984,0.8984,1) |  |
| T2WI | 4500ms | 105ms | 5mm | 320×230 | 176*220mm | 150° | (0.4911,0.4911,6.5) |  |
| FLAIR | 6000ms | 81ms | 6mm | 320×196 | 195*220mm | 150° | (0.6875,0.6875,6.5) |  |
| DWI | 5300ms | 102ms | 5mm | 192×192 | 230*230mm | 90° | (0.5729,0.5729,6.5) | b=0, 1000s/mm2 |

* Using 0.1 mmol/kg bodyweight of gadobenate dimeglumine [Multihance, Braccosine, Shanghai, China]. It was administered intravenously at a rate of 4.0 ml/s, followed by a 30-ml saline flush

**Table. S2. The list of all original features.**

| **Matrixes** | **Features^a^** |
| --- | --- |
| First order | 1. Energy |
|  | 2. Total Energy |
|  | 3. Entropy |
|  | 4. Minimum |
|  | 5. 10th percentile |
|  | 6. 90th percentile |
|  | 7. Maximum |
|  | 8. Mean |
|  | 9. Median |
|  | 10. Interquartile Range |
|  | 11. Range |
|  | 12. Mean Absolute Deviation |
|  | 13. Robust Mean Absolute Deviation |
|  | 14. Root Mean Squared |
|  | 15. Skewness |
|  | 16. Kurtosis |
|  | 17. Variance |
|  | 18. Uniformity |
| Shape | 1. Mesh Volume |
|  | 2. Voxel Volume |
|  | 3. Surface Area |
|  | 4. Surface Area to Volume ratio |
|  | 5. Sphericity |
|  | 6. Maximum 3D diameter |
|  | 7. Maximum 2D diameter (Slice) |
|  | 8. Maximum 2D diameter (Column) |
|  | 9. Maximum 2D diameter (Row) |
|  | 10. Major Axis Length |
|  | 11. Minor Axis Length |
|  | 12. Least Axis Length |
|  | 13. Elongation |
|  | 14. Flatness |
| Gray Level Co-occurrence Matrix (GLCM) | 1. Autocorrelation |
|  | 2. Joint Average |
|  | 3. Cluster Prominence |
|  | 4. Cluster Shade |
|  | 5. Cluster Tendency |
|  | 6. Contrast |
|  | 7. Correlation |
|  | 8. Difference Average |
|  | 9. Difference Entropy |
|  | 10. Difference Variance |
|  | 11. Joint Energy |
|  | 12. Joint Entropy |
|  | 13. Informational Measure of Correlation (IMC) 1 |
|  | 14. Informational Measure of Correlation (IMC) 2 |
|  | 15. Inverse Difference Moment (IDM) |
|  | 16. Inverse Difference Moment Normalized (IDMN) |
|  | 17. Inverse Difference (ID) |
|  | 18. Inverse Difference Normalized (IDN) |
|  | 19. Inverse Variance |
|  | 20. Maximum Probability |
|  | 21. Sum Entropy |
|  | 22. Sum of Squares |
| Gray Level Run Length Matrix (GLRLM) | 1. Short Run Emphasis (SRE) |
|  | 2. Long Run Emphasis (LRE) |
|  | 3. Gray Level Non-Uniformity (GLN) |
|  | 4. Gray Level Non-Uniformity Normalized (GLNN) |
|  | 5. Run Length Non-Uniformity (RLN) |
|  | 6. Run Length Non-Uniformity Normalized (RLNN) |
|  | 7. Run Percentage (RP) |
|  | 8. Gray Level Variance (GLV) |
|  | 9. Run Variance (RV) |
|  | 10. Run Entropy (RE) |
|  | 11. Low Gray Level Run Emphasis (LGLRE) |
|  | 12. High Gray Level Run Emphasis (HGLRE) |
|  | 13. Short Run Low Gray Level Emphasis (SRLGLE) |
|  | 14. Short Run High Gray Level Emphasis (SRHGLE) |
|  | 15. Long Run Low Gray Level Emphasis (LRLGLE) |
|  | 16. Long Run High Gray Level Emphasis (LRHGLE) |
| Gray Level Size Zone Matrix (GLSZM) | 1. Small Area Emphasis (SAE) |
|  | 2. Large Area Emphasis (LAE) |
|  | 3. Gray Level Non-Uniformity (GLN) |
|  | 4. Gray Level Non-Uniformity Normalized (GLNN) |
|  | 5. Size-Zone Non-Uniformity (SZN) |
|  | 6. Size-Zone Non-Uniformity Normalized (SZNN) |
|  | 7. Zone Percentage (ZP) |
|  | 8. Gray Level Variance (GLV) |
|  | 9. Zone Variance (ZV) |
|  | 10. Zone Entropy (ZE) |
|  | 11. Low Gray Level Zone Emphasis (LGLZE) |
|  | 12. High Gray Level Zone Emphasis (HGLZE) |
|  | 13. Small Area Low Gray Level Emphasis (SALGLE) |
|  | 14. Small Area High Gray Level Emphasis (SAHGLE) |
|  | 15. Large Area Low Gray Level Emphasis (LALGLE) |
|  | 16. Large Area High Gray Level Emphasis (LAHGLE) |
| Neigbouring Gray Tone Difference Matrix (NGTDM) | 1. Coarseness |
|  | 2. Contrast |
|  | 3. Busyness |
|  | 4. Complexity |
|  | 5. Strength |
| Gray Level Dependence Matrix (GLDM) | 1. Small Dependence Emphasis (SDE) |
|  | 2. Large Dependence Emphasis (LDE) |
|  | 3. Gray Level Non-Uniformity (GLN) |
|  | 4. Dependence Non-Uniformity (DN) |
|  | 5. Dependence Non-Uniformity Normalized (DNN) |
|  | 6. Gray Level Variance (GLV) |
|  | 7. Dependence Variance (DV) |
|  | 8. Dependence Entropy (DE) |
|  | 9. Low Gray Level Emphasis (LGLE) |
|  | 10. High Gray Level Emphasis (HGLE) |
|  | 11. Small Dependence Low Gray Level Emphasis (SDLGLE) |
|  | 12. Small Dependence High Gray Level Emphasis (SDHGLE) |
|  | 13. Large Dependence Low Gray Level Emphasis (LDLGLE) |
|  | 14. Large Dependence High Gray Level Emphasis (LDHGLE) |

a. The formula of features was in https://pyradiomics.readthedocs.io/en/latest/.

**Table. S3. Radiological characteristics between the two groups of IDH/pTERT glioma**

|  | Training and test set | | | Independent validation set | | |  |
| --- | --- | --- | --- | --- | --- | --- | --- |
| Variables | **IDHmut/pTERTmut(N=22)** | **Non-IDHmut/pTERTmut (N=118)** | **P value^a^** | **IDHmut/pTERTmut(N=7)** | **Non-IDHmut/pTERTmut (N=27)** | **P value^b^** | **P value^c^** |
| Enhancement Quality |  |  | <.001* |  |  | 0.136* | 0.079* |
| Non Enhancing | 17 | 34 |  | 5 | 12 |  |  |
| Minimal/Mild Enhancing | 4 | 38 |  | 1 | 4 |  |  |
| Marked/Avid Enhancing | 1 | 46 |  | 0 | 40 |  |  |
| T2/FLAIR Mismatch |  |  | 0.205* |  |  | 0.567* | 0.517* |
| Yes | 1 | 17 |  | 1 | 2 |  |  |
| No | 21 | 101 |  | 5 | 24 |  |  |

~~*~~ Chi-square test

a. The characteristics of the two groups (IDHmut/pTERTmut and non-IDHmut/pTERTmut) in training/test cohort were compared using the chi-square test.

b. The characteristics of the two groups (IDHmut/pTERTmut and non-IDHmut/pTERTmut) in independent validation cohort were compared using the chi-square test.

c. The characteristics of independent validation cohort and training/test cohort were compared using the chi-square test.

**Table. S4. Thirty-nine selected features after LASSO feature dimensionality reduction**

| **Image_type** | **Fearure_type** | **Selected features** | **Lasso coeffcient** | **FDR Adj p (kruskal wallis test)^a^** | **FDR Adj p (Mann-whitney test)^b^** | **FDR p (Mann-whitney test)^c^** |
| --- | --- | --- | --- | --- | --- | --- |
| LoG | firstorder | ADC_log-sigma-5-0-mm-3D_firstorder_RootMeanSquared | -1.66E-02 | 3.01E-07 | 2.33E-04 | 5.39E-01 |
| wavelwt | texture | t1Gd_wavelet-LLL_glszm_SmallAreaEmphasis | -9.28E-03 | 2.07E-05 | 1.20E-04 | 4.10E-02 |
| wavelwt | texture | t1Gd_wavelet-HHH_gldm_LargeDependenceEmphasis | 1.19E-02 | 3.09E-05 | 2.33E-04 | 1.66E-01 |
| wavelwt | texture | t1Gd_wavelet-HLH_glcm_Imc2 | -2.71E-02 | 5.90E-05 | 2.33E-04 | 1.40E-01 |
| LoG | texture | t1Gd_log-sigma-5-0-mm-3D_glcm_Imc1 | -3.62E-02 | 6.90E-05 | 1.87E-04 | 4.10E-02 |
| LoG | texture | t1Gd_log-sigma-5-0-mm-3D_glszm_LargeAreaLowGrayLevelEmphasis | 1.70E-02 | 1.90E-04 | 2.54E-04 | 8.49E-02 |
| LoG | texture | t1Gd_log-sigma-4-0-mm-3D_glszm_GrayLevelNonUniformityNormalized | 3.07E-02 | 1.90E-04 | 2.54E-04 | 4.62E-02 |
| original | texture | t1Gd_original_glcm_Correlation | 9.81E-03 | 1.99E-04 | 1.22E-03 | 3.21E-01 |
| wavelwt | texture | flair_wavelet-LLL_gldm_DependenceVariance | -2.82E-02 | 1.99E-04 | 3.45E-04 | 1.02E-01 |
| wavelwt | texture | flair_wavelet-LLH_gldm_DependenceVariance | 5.63E-04 | 5.96E-04 | 2.08E-03 | 1.23E-01 |
| wavelwt | texture | flair_wavelet-LLL_gldm_SmallDependenceHighGrayLevelEmphasis | 5.14E-02 | 1.21E-03 | 1.22E-03 | 1.02E-01 |
| LoG | texture | flair_log-sigma-2-0-mm-3D_gldm_DependenceVariance | -4.45E-02 | 2.63E-03 | 1.59E-03 | 8.49E-02 |
| wavelwt | firstorder | t1Gd_wavelet-LHL_firstorder_Range | -8.73E-03 | 6.53E-03 | 1.22E-03 | 1.02E-01 |
| wavelwt | texture | ADC_wavelet-LHH_glcm_Correlation | -1.47E-02 | 6.93E-03 | 5.32E-03 | 1.02E-01 |
| wavelwt | texture | flair_wavelet-HLH_glszm_GrayLevelNonUniformityNormalized | -3.03E-02 | 8.64E-03 | 2.75E-03 | 1.06E-02 |
| LoG | texture | t1Gd_log-sigma-2-0-mm-3D_glcm_Imc2 | -2.57E-02 | 1.10E-02 | 2.08E-03 | 4.10E-02 |
| wavelwt | texture | ADC_wavelet-HHH_glcm_Imc1 | -1.07E-02 | 1.45E-02 | 2.96E-03 | 4.62E-02 |
| original | shape | t1Gd_original_shape_Sphericity | 1.82E-07 | 2.43E-02 | 1.52E-02 | 3.21E-01 |
| original | shape | flair_original_shape_Sphericity | 6.32E-03 | 2.43E-02 | 1.52E-02 | 3.21E-01 |
| original | shape | ADC_original_shape_Sphericity | 1.05E-04 | 2.43E-02 | 1.52E-02 | 3.21E-01 |
| wavelwt | texture | t1Gd_wavelet-LLL_glszm_ZoneVariance | -1.67E-02 | 3.03E-02 | 3.90E-01 | 3.21E-01 |
| wavelwt | texture | flair_wavelet-HLH_glszm_SmallAreaEmphasis | 6.94E-03 | 4.33E-02 | 5.32E-03 | 4.62E-02 |
| wavelwt | texture | flair_wavelet-HLH_glszm_SizeZoneNonUniformityNormalized | 8.79E-03 | 5.08E-02 | 7.05E-03 | 4.62E-02 |
| wavelwt | texture | ADC_wavelet-LHH_glszm_LargeAreaLowGrayLevelEmphasis | -2.55E-03 | 5.10E-02 | 8.53E-03 | 1.02E-01 |
| wavelwt | texture | flair_wavelet-HHL_glszm_GrayLevelVariance | 1.34E-02 | 5.10E-02 | 8.19E-03 | 4.10E-02 |
| wavelwt | firstorder | flair_wavelet-HLL_firstorder_Maximum | 2.11E-02 | 5.10E-02 | 1.80E-02 | 4.10E-02 |
| wavelwt | firstorder | ADC_wavelet-LHL_firstorder_Maximum | 1.87E-02 | 5.90E-02 | 4.40E-02 | 6.27E-01 |
| wavelwt | texture | t1Gd_wavelet-HHH_glszm_LowGrayLevelZoneEmphasis | 3.30E-02 | 8.50E-02 | 9.00E-02 | 5.67E-01 |
| wavelwt | firstorder | flair_wavelet-HHL_firstorder_Median | -3.36E-02 | 1.37E-01 | 4.96E-02 | 1.59E-01 |
| wavelwt | texture | flair_wavelet-HHL_glszm_SmallAreaHighGrayLevelEmphasis | 1.20E-02 | 1.44E-01 | 3.95E-02 | 4.62E-02 |
| wavelwt | firstorder | flair_wavelet-HLH_firstorder_Median | 1.86E-02 | 2.15E-01 | 2.59E-01 | 2.79E-01 |
| wavelwt | texture | flair_wavelet-HHH_glszm_SmallAreaEmphasis | -6.92E-03 | 3.35E-01 | 4.28E-01 | 2.79E-01 |
| original | texture | ADC_original_glcm_Imc1 | 1.51E-02 | 3.35E-01 | 1.86E-01 | 1.59E-01 |
| wavelwt | texture | ADC_wavelet-LLL_glszm_LargeAreaLowGrayLevelEmphasis | 9.46E-03 | 3.38E-01 | 9.77E-01 | 5.13E-01 |
| wavelwt | texture | ADC_wavelet-HHL_gldm_SmallDependenceHighGrayLevelEmphasis | 4.87E-03 | 3.64E-01 | 2.71E-01 | 8.19E-01 |
| LoG | texture | flair_log-sigma-5-0-mm-3D_glcm_ClusterShade | 1.41E-02 | 5.47E-01 | 4.28E-01 | 5.39E-01 |
| wavelwt | firstorder | flair_wavelet-HLH_firstorder_Skewness | -1.73E-02 | 6.12E-01 | 6.56E-01 | 8.75E-01 |
| wavelwt | firstorder | flair_wavelet-HHH_firstorder_Skewness | 1.63E-02 | 7.54E-01 | 4.40E-01 | 8.75E-01 |
| wavelwt | firstorder | ADC_wavelet-HHH_firstorder_Skewness | 1.25E-02 | 9.29E-01 | 9.86E-01 | 9.18E-01 |

a. P-value of comparison among four subgroups (IDHmut pTERTmut, IDHmut pTERTwt, IDHwt pTERTmut, and IDHwt pTERTwt).

b. P-value of comparison between IDHmut/pTERTmut and Non-IDHmut/pTERTmut groups.

c. P-value of comparison between IDHmut/pTERTmut and IDHmut/pTERTwt groups.

**Table. S5. The raw data of radiomic features**

Raw data table for all extracted radiomic features in three sequences.

**Figure. S1.** **Feature selection by LASSO feature dimensionality reduction.**

**A.** λ **selection by MSE**


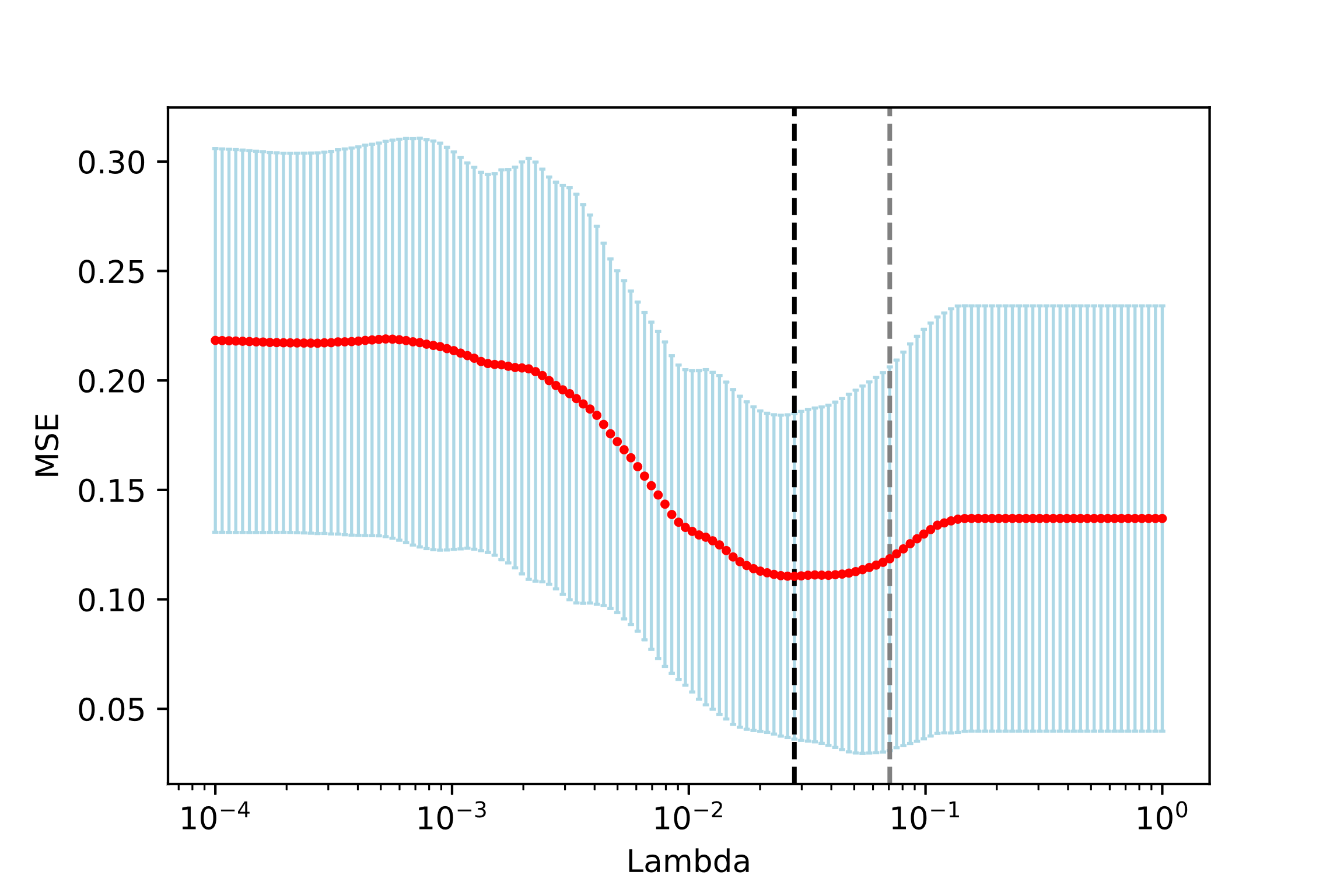


**
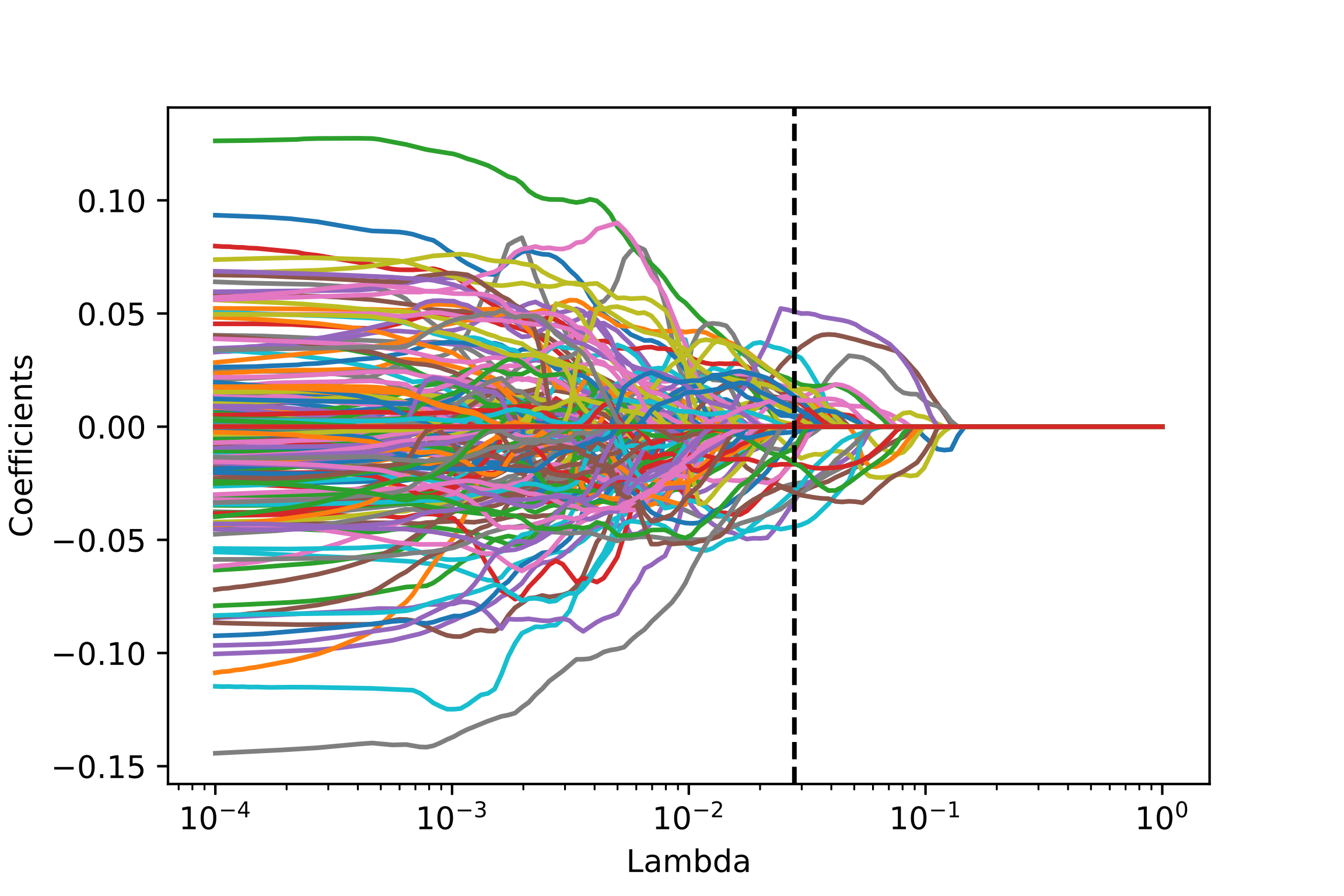
B. Feature selection by coefficient**

A and B, Feature selection using the least absolute shrinkage and selection operator (LASSO) binary logistic regression model. A, The MSE was plotted vs. log(λ). The vertical black line represented the chosen parameter. B, Colored lines represented the coefficient of each feature, and the vertical black line was set at the selected λ, where 39 features had non-zero coefficients.

Abbreviations: LASSO, the least absolute shrinkage and selection operator.

**Figure. S2. Correlation between features**


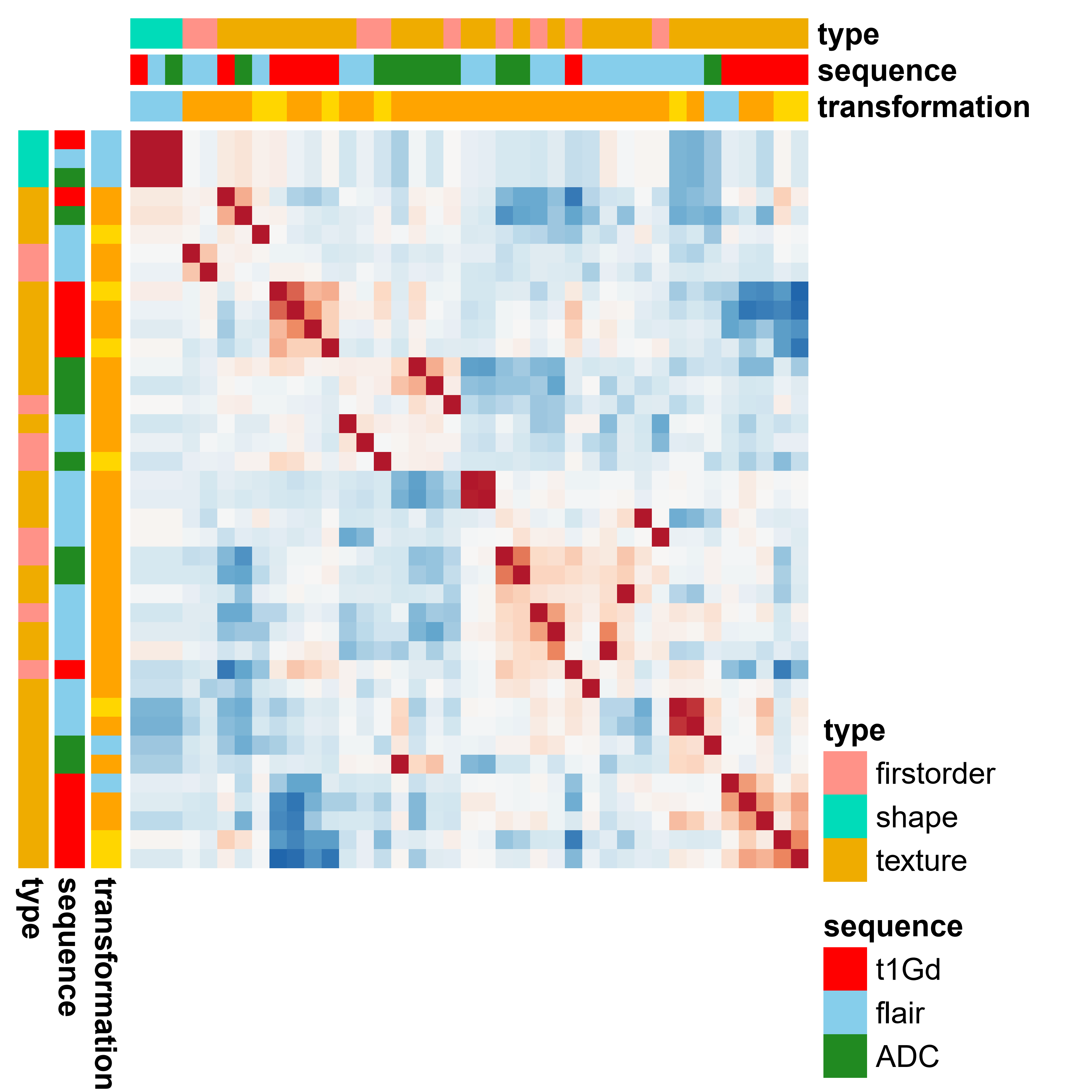


Figure. S2. Clustering features found a higher correlation between the extracted features from original images than those extracted from LoG and wavelet transformed images

**Figure. S3.** Pearson correlation of features before and after LASSO.

**A.** Before LASSO


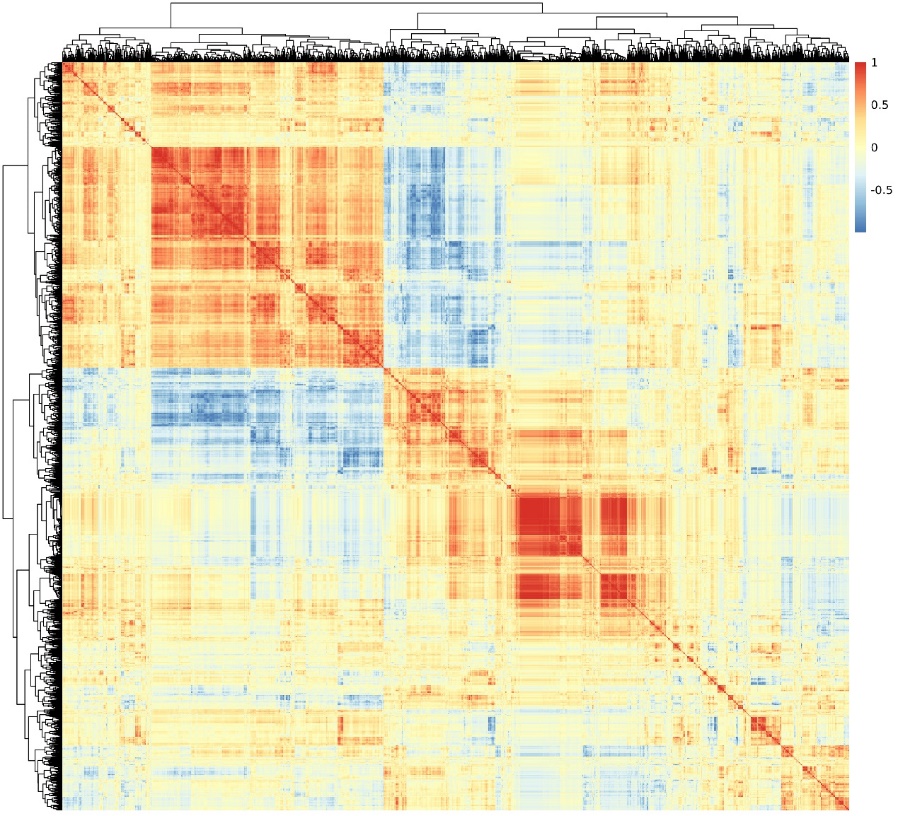


**B.** After LASSO


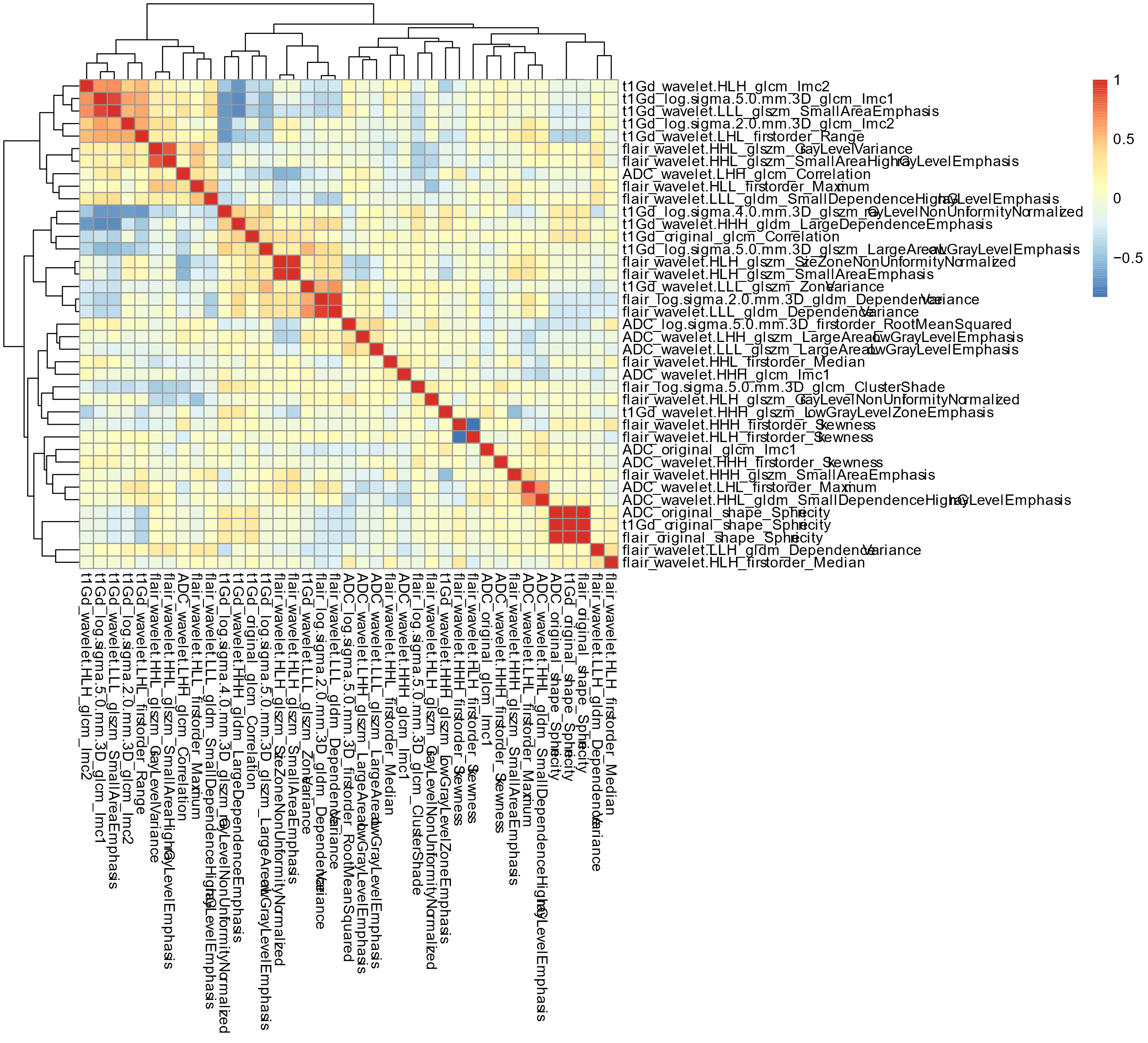


**Figure. S4. A boxplot comparing the difference in key radiomics features between IDHmut/pTERTmut and IDHmut/pTERTwt groups.**


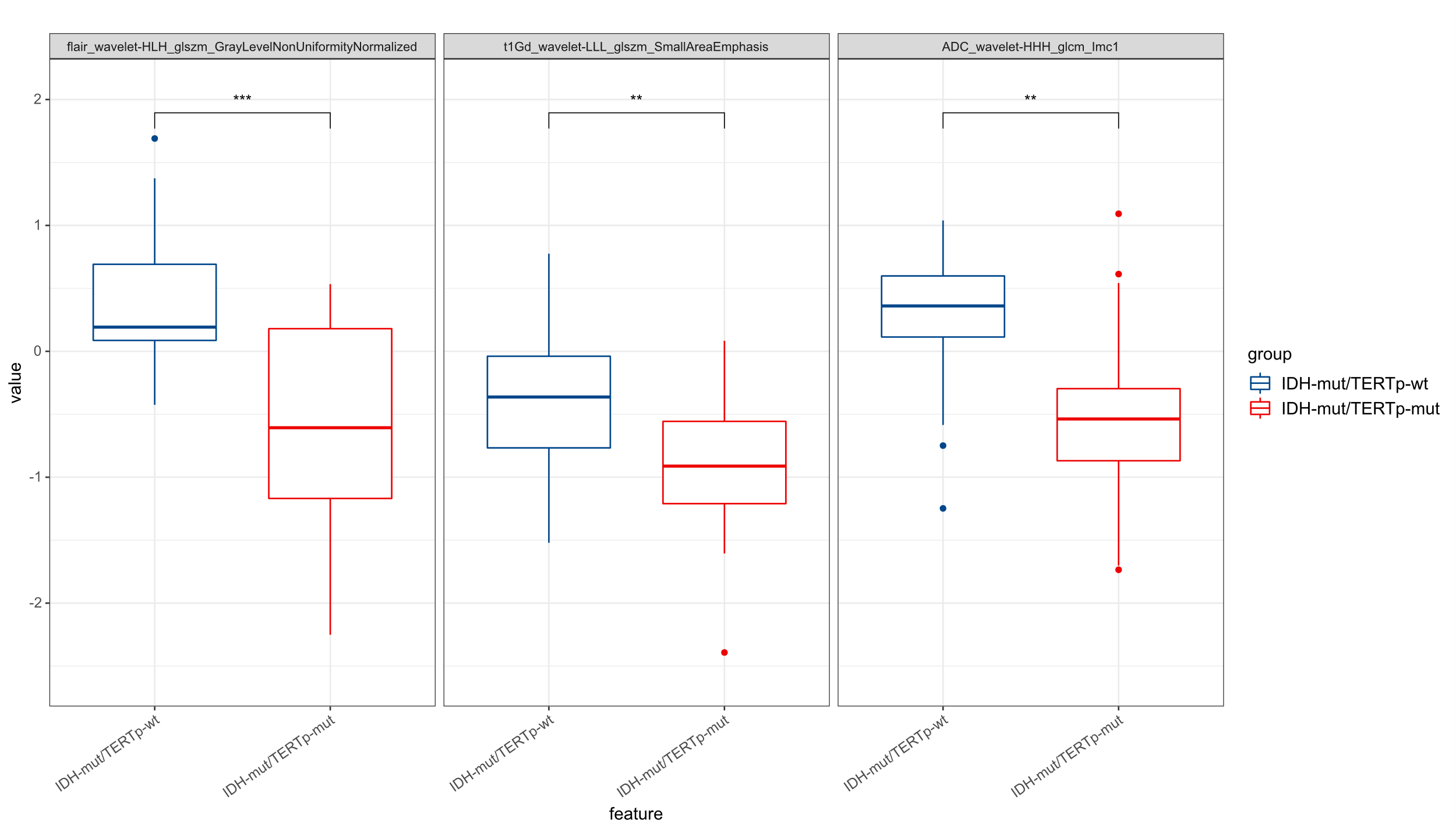


Figure. S3. Boxplot of feature values. The value of key radiomics features in each sequence between IDHmut/pTERTmut and IDHmut/pTERTwt groups were compared using the Mann-Whitney test

**Figure. S5. The appearance of MR image corresponds to 3 radiomics features in IDHmut/pTERTmut and IDHmut/pTERTwt gliomas.**

**A. Visual characteristic of T1Gd_wavelet-LLL_glszm_SmallAreaEmphasis（SAE）**

~~
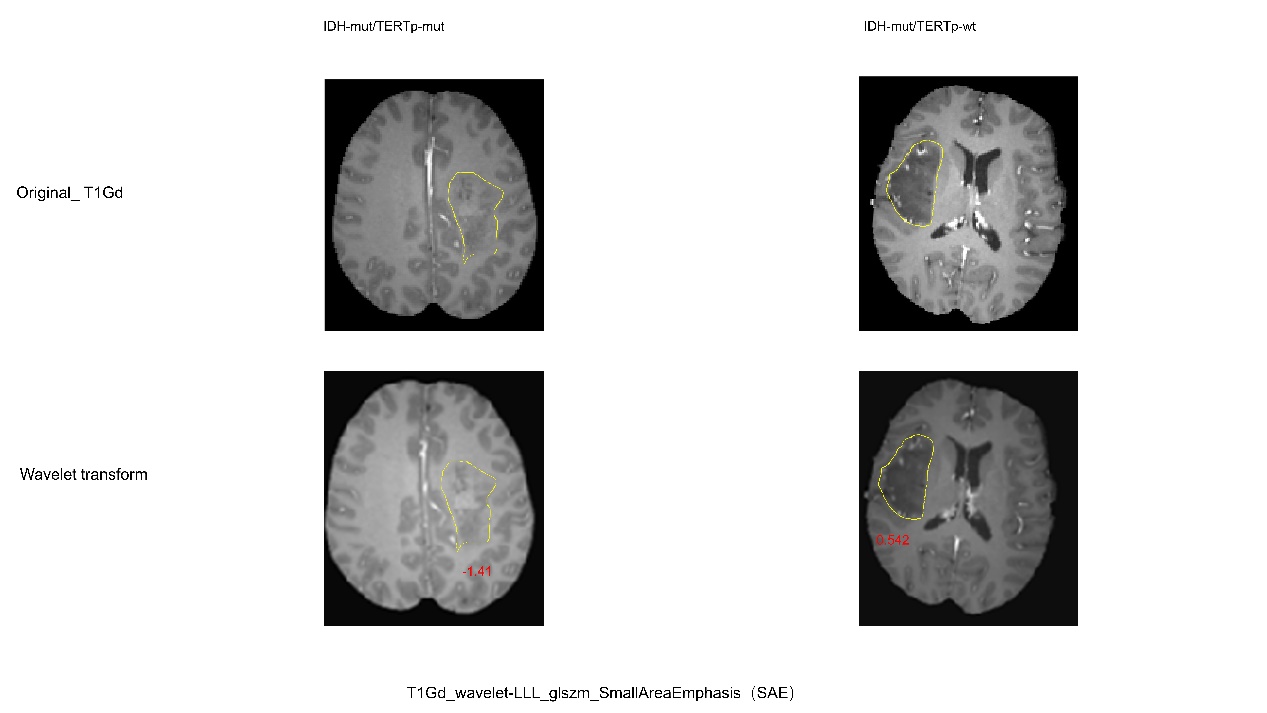
~~

**B. Visual characteristic of FLAIR_wavelet-HLH_glszm_GrayLevelNonUniformityNormalized（GLNN）**

~~
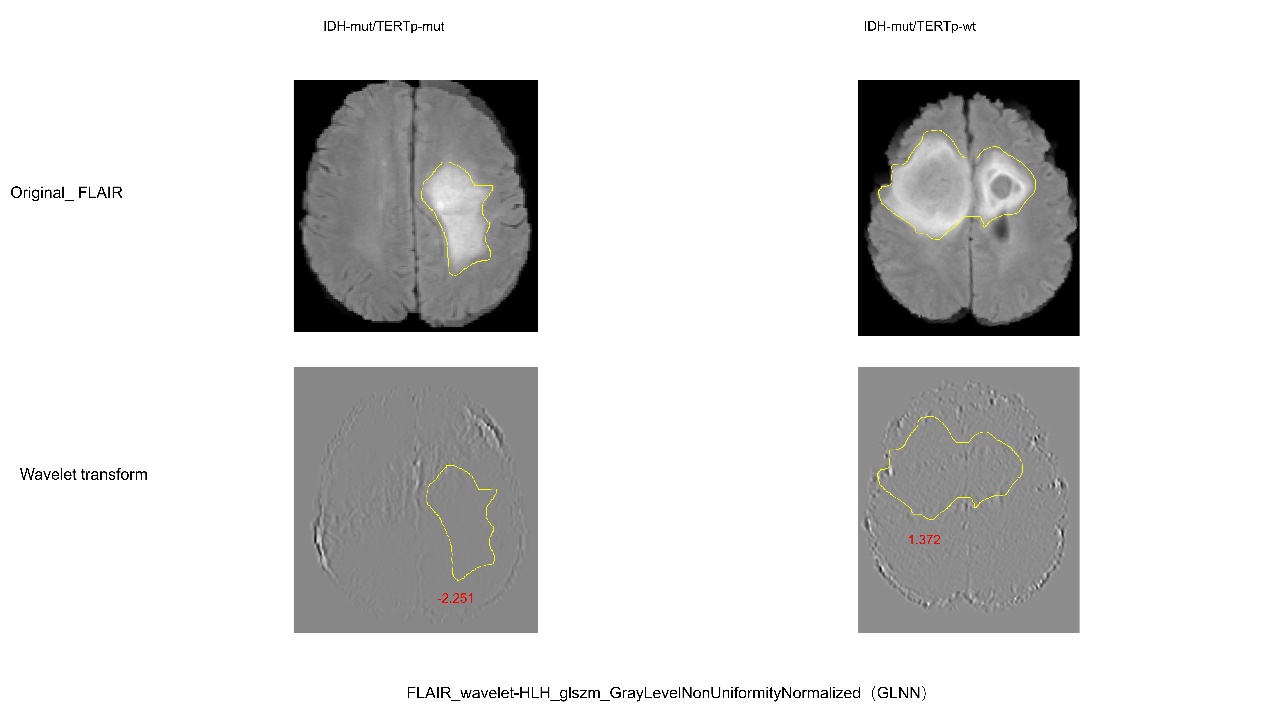
~~

**C. Visual characteristic of ADC_wavelet-HHH_glcm_Imc1**

~~
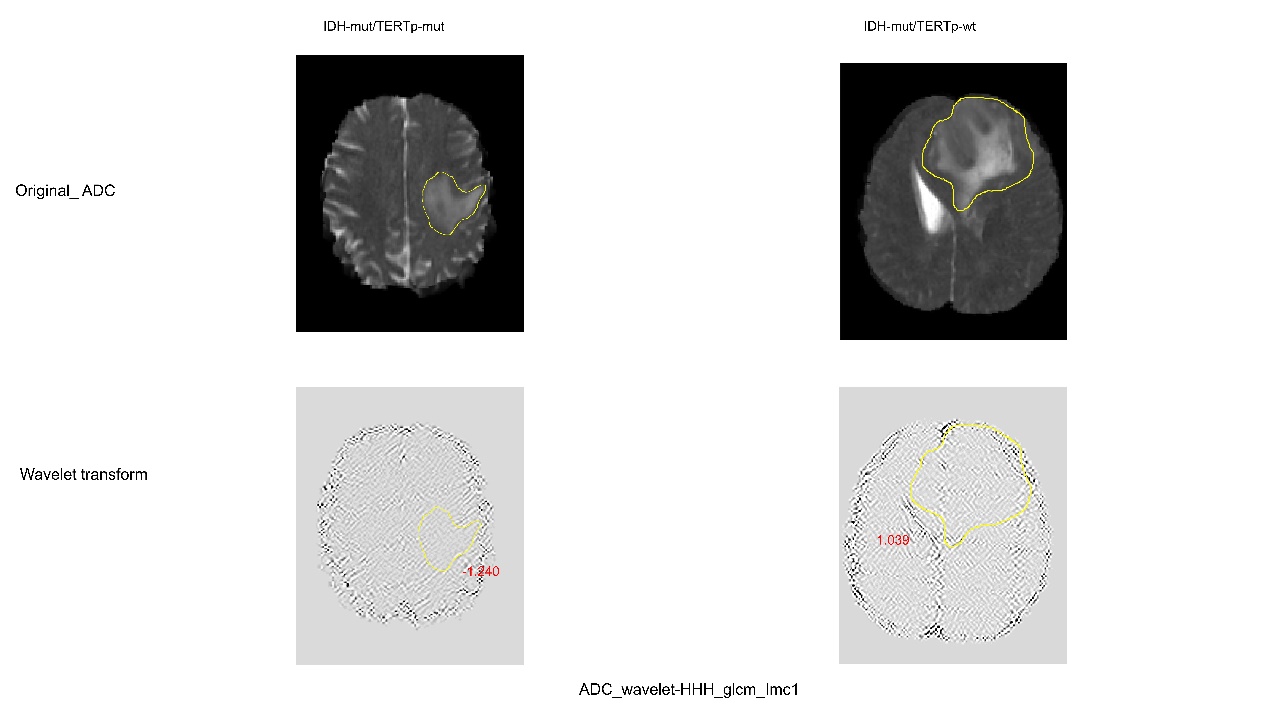
~~

The FLAIR and ADC differential features could be interpreted into simple or uniform intensity signals in the high-pass wavelet filter transformed images, while the t1Gd_wavelet-LLL_glszm_SmallAreaEmphasis indicated fewer fine patterns (small-size connected voxel zones) in the smoothed T1c image. Yellow lines indicated the region of gliomas. Red numbers denoted the feature values of ROI (region of interest).

Abbreviations: Imc1: Informational Measure of Correlation.

Reference：

1 Burt P, Adelson E (1983) The Laplacian Pyramid as a Compact Image Code. IEEE Transactions on Communications 31:532-540

2 Benoit-Cattin H, Baskurt A, Turjman F, Prost R (1997) 3D medical image coding using separable 3D wavelet decomposition and lattice vector quantization. Signal Processing 59:139-153
